# Impacts of reference population size and methods on the accuracy of genomic prediction for fleece traits in Inner Mongolia Cashmere Goats

**DOI:** 10.3389/fvets.2024.1325831

**Published:** 2024-02-05

**Authors:** Xiaochun Yan, Jiaxin Zhang, Jinquan Li, Na Wang, Rui Su, Zhiying Wang

**Affiliations:** ^1^College of Animal Science, Inner Mongolia Agricultural University, Hohhot, China; ^2^Inner Mongolia Key Laboratory of Sheep and Goat Genetics Breeding and Reproduction, Hohhot, China; ^3^Key Laboratory of Mutton Sheep and Goat Genetics and Breeding, Ministry of Agriculture And Rural Affairs, Hohhot, China; ^4^Engineering Research Centre for Goat Genetics and Breeding, Inner Mongolia Autonomous Region, Hohhot, China; ^5^Inner Mongolia Yiwei White Cashmere Goat Co., Ltd., Hohhot, China

**Keywords:** genomic selection, Inner Mongolia Cashmere Goats, GBLUP method, Bayesian method, reference population sizes

## Abstract

**Introduction:**

Inner Mongolia Cashmere Goats (IMCGs) are famous for its cashmere quality and it’s a unique genetic resource in China. Therefore, it is necessary to use genomic selection to improve the accuracy of selection for fleece traits in Inner Mongolia cashmere goats. The aim of this study was to determine the effect of methods (GBLUP, BayesA, BayesB, Bayesian LASSO, Bayesian Ridge Region) and the reference population size on accuracy of genomic selection in IMCGs.

**Methods:**

This study fully utilizes the pedigree and phenotype records of fleece traits in 2255 individuals, genotype of 50794 SNPs after quality control, and environmental data to perform genomic selection of fleece traits. Then GBLUP and Bayes series methods (BayesA, BayesB, Bayesian LASSO, Bayesian Ridge Region) were used to perform estimates of genetic parameter and genomic breeding value. And the accuracy of genomic estimated breeding value (GEBV) is evaluated using the five-fold cross validation method. And the analysis of variance and multiple comparison methods were used to determine the best method for genomic selection in fleece traits of IMCGs. Further the different reference population sizes (500, 1000, 1500, and 2000) was set. Then the best method was applied to estimate genome breeding values, and evaluate the impact of reference population sizes on the accuracy of genome selection for fleece traits in IMCGs.

**Results:**

It was found that the genomic prediction accuracy for each fleece trait in IMCGs by GBLUP method is highest, and it is significantly higher than that obtained by Bayesian method. The accuracy of breeding value estimation is 58.52% -68.49%. Also, it was found that the size of the reference population has a significant impact on the accuracy of genome prediction of fleece traits. When the reference population size is 2000, the accuracy of genomic prediction for each fleece trait is significantly higher than other levels, with accuracy of 55.47% -67.87%. This provides a theoretical basis for design a reasonable genome selection plan for Inner Mongolia cashmere goats in the later stag.

## 1 Introduction

China is a large country in terms of the number of cashmere goats and cashmere production in the world. By the end of 2022, the number of goats in China was 92.0 million, and the cashmere production was 15243.64 tons (http://www.stats.gov.cn), which accounts for 80% of the world’s goat population (https://www.fao.org/). Inner Mongolia Cashmere Goats (IMCGs) are a major cashmere goat breed in China, which is famous for its high cashmere production and excellent quality of cashmere. According to geographical distribution, it is divided into three types, namely, Arbas type, Erlangshan type, and Alxa type (1). Methods to reduce cashmere diameter (CD) and increase cashmere production (CP) are important projects of Inner Mongolia Cashmere Goats breeding. In previous studies, genetic evaluation for fleece traits in IMCGs was performed by the BLUP method (2). The fleece traits had a certain degree of improvement. With the development of quantitative genetics and molecular biology, the breeding methods of livestock have improved (3). In order to improve goat efficiency and achieve early selection, the breeding methods of goats should be updated. Therefore, genomic selection needs to be performed. The idea of genomic selection was proposed by Meuwissen et al (4). It had been reported that genomic selection has significant advantages in traits with low habitability and which are difficult to measure (5). It was confirmed that genomic selection can improve the accuracy of estimated breeding values, increase genetic progress, and reduce breeding costs (6–8). The factors that affect the accuracy of genomic selection include methods (9), reference population size (10), heritability (11), and marker density (12).

With the development of genetics and statistics, a large number of methods for estimating genomic breeding values have been continuously proposed. According to different statistical models, genomic breeding value estimation methods can be divided into three categories: genome best linear unbiased prediction (GBLUP), ridge regression best linear unbiased prediction (RRBLUP), and Bayesian series methods (BayesA, BayesB, Bayes Cp, Bayes LASSO, and BayesRR). The GBLUP and RRBLUP models assume that the variance explained by each SNP is equal, and the advantage of this assumption is that only one variance needs to be estimated. In actualality, the SNP effects have different variance structures. Peters used different BayesB models to compare the accuracy of GEBV for milk traits of 695 Canadian Holstein cows (13). It was shown that the prediction accuracy with the BayesB method was significantly higher than that using the GBLUP method for milk traits. Lopes used five methods, including BayesA, BayesB, Bayes C π , BLUP, and SSGBLUP, to evaluate the accuracy of genomic prediction for meat and carcass traits in Nelore cattle. It was found that the accuracy of GEBV among the five methods had no significant difference (14).

Generally, the larger the reference population size, the richer the genotype data and phenotype information, and the higher the accuracy of GEBV obtained (15). Takeda et al. compared the estimated breeding values for five carcass traits of Japanese black cattle under different reference population sizes (16). It was found that the accuracy of GEBV was increasing as the reference population size expanded. Lillehammer et al. used simulated data to perform genomic selection of maternal traits in pigs. It was illustrated that the genetic progress obtained by the reference population size of 1,000 was significantly higher than that in the 5,000 reference population (17).

The implementation of genomic selection for cashmere goats in China is relatively late. Previous studies have identified factors that affect the accuracy of GEBV in goats using simulated data. It is the first time to perform a genomic selection of the fleece traits in Inner Mongolia Cashmere Goats. This study used five different methods to estimate the genomic breeding values of fleece traits in IMCGs and compared the impact of these methods on the accuracy of GEBV. Then, the best methods were used to determine the impact of reference population size on the accuracy of GEBV, providing a theoretical basis for designing the breeding plan for fleece traits in Inner Mongolia Cashmere Goats.

## 2 Materials and methods

### 2.1 Genotype data

The individuals were genotyped using the Illumina GGP_Goat_70K BeadChip (Illumina, San Diego, CA). Markers on the X chromosome were discarded. SNPs were performed as quality control based on minor allele frequency (MAF > 0.05), proportion of missing genotypes (missing<0.05), and Hardy–Weinberg equilibrium (HWE > 10^−6^). Unqualified SNPs were removed. Moreover, individuals with more than 10% missing genotypes were excluded. In this study, 44 individuals and 16,294 SNPs were deleted from the raw genotype data. Finally, 2,255 individuals and 50,794 SNPs were used in the next analysis.

### 2.2 Phenotypic data

The phenotypic data were collected from Inner Mongolia Yiwei White Cashmere Goat Limited Liability Company, Wulan Town, Etuoke Banner, Ordos City, Inner Mongolia Autonomous Region, China (39°12′N; 107°97′E). In this study, the production performance records of fleece traits for 2,255 individuals (372 males and 1883 females) at ages 1 to 3 were collected from 2018 to 2021. The four fleece traits, including cashmere production (CP), cashmere diameter (CD), cashmere length (CL), and fiber length (FL), were considered in this study. The basic statistics of phenotype data were analyzed using Microsoft Excel and R software.

### 2.3 Estimation of genomic breeding value

In this study, the fixed effects, including sex, year of production, herd, and individual age, were considered. They were determined based on the previous results of our research team (2, 18–20). The linear mixed model was used to estimate the genomic breeding values for fleece traits in IMCGs with BayesA, BayesB, Bayesian LASSO, Bayesian Ridge Regression, and GBLUP methods. All methods were performed by the BGLR software (21).

#### 2.3.1 GBLUP method

Van Raden (22) proposed the GBLUP method, which uses the additive effect matrix G constructed by genetic markers to replace the traditional kinship matrix A constructed by pedigree and then estimates the genomic breeding value of individuals. The model for the GBLUP method is as follows (Eq. 1):


(1) y=μ+Xb+Za+e

where y is the vector of the observations, μ is the mean value vector of the observations, b is the vector of fixed effects, a is a vector of additive genetic effects, following a normal distribution of $a\sim N\left(0,G\sigma_{a}^{2}\right)$ , in which $\sigma_{a}^{2}$ is the variance of additive genetic effect, and e is a vector of residual. The matrix X is the incidence matrix for the fixed effects and Z is the incidence matrix for additive genetic effects.

#### 2.3.2 Bayesian series methods

The BayesA method assumes that a large number of markers have a smaller effect on the target trait, while a small number of markers have a larger effect and follow t-distribution. The BayesB method assumes that some SNP effects also follow t-distribution, but a large number of effects are zero, only some QTLs have a larger effect. Bayes Lasso is the same as BayesA, but the difference between them is that it assumes that the marker effect follows a double exponential distribution, resulting in a corresponding change in the posterior distribution of the labeling effect. The Bayesian Ridge Region (BayesRR) method assumes that the variance effect of each locus is specified by a certain percentage of the total genetic variance. The effects of the locus for BayesRR follow multiple normal distributions. The hypothetical distribution of all the effects of the marker in each Bayesian method and the formula of effect distribution are shown in Table 1 (6, 23–25). In this study, the model of Bayes methods is as follows (Eq. 2):


(2) $$y=\mu +Xb+\overset{n}{\underset{j}{\sum }}\left(Z_{ij}a_{j}\right)+e$$

**Table 1:** Basic description of Bayesian methods.

Methods	Presenter	Assumed distribution of effect	Unknown parameter
Bayes A	Meuwissen et al. (4)	*t*	–
Bayes B	Meuwissen et al. (23)	Point-*t*	–
Bayesian LASSO	Park and Casella (24)	Double exponential	λt
BayesRR	Brøndum et al. (25)	Multiple normal	γ

Here, y is the vector of the observations, μ is the mean value vector of the observations, X is the incidence matrix for the fixed effects, and b is the vector of fixed effects. $Z_{ij}$ represents the genotype of the individual i at site j and $a_{j}$ represents the effect value of the site j , and therefore $\overset{n}{\underset{j}{\sum }}\left(Z_{ij}a_{j}\right)$ refers to the breeding value corresponding to the individual i , e to the vector of residual effects.

### 2.4 Accuracy of predicted genomic breeding value

In this study, 5-fold cross-validation was used to evaluate the accuracy of genomic prediction. First, the 2,255 individuals were randomly divided into five groups, and then one group (451 individuals) was selected as the validation population at each time, and the other four groups (1804 individuals) were used as the training population. The five repetitions are executed. The accuracy of genomic prediction is evaluated by calculating correlation coefficients between GEBV and the true corrected phenotype value in the validation population.

Finally, we used a one-way analysis of variance and multiple comparison methods to determine the best method for genomic selection of the fleece traits of IMCGs. Furthermore, different reference population sizes (500, 1,000, 1,500, and 2,000) were set, and then the best method was used to estimate GEBV and to evaluate the impact of reference population sizes on the accuracy of genomic prediction for fleece traits in IMCGs.

## 3 Results

### 3.1 Genotypic characteristics and phenotypic statistics

**Figure 1:**
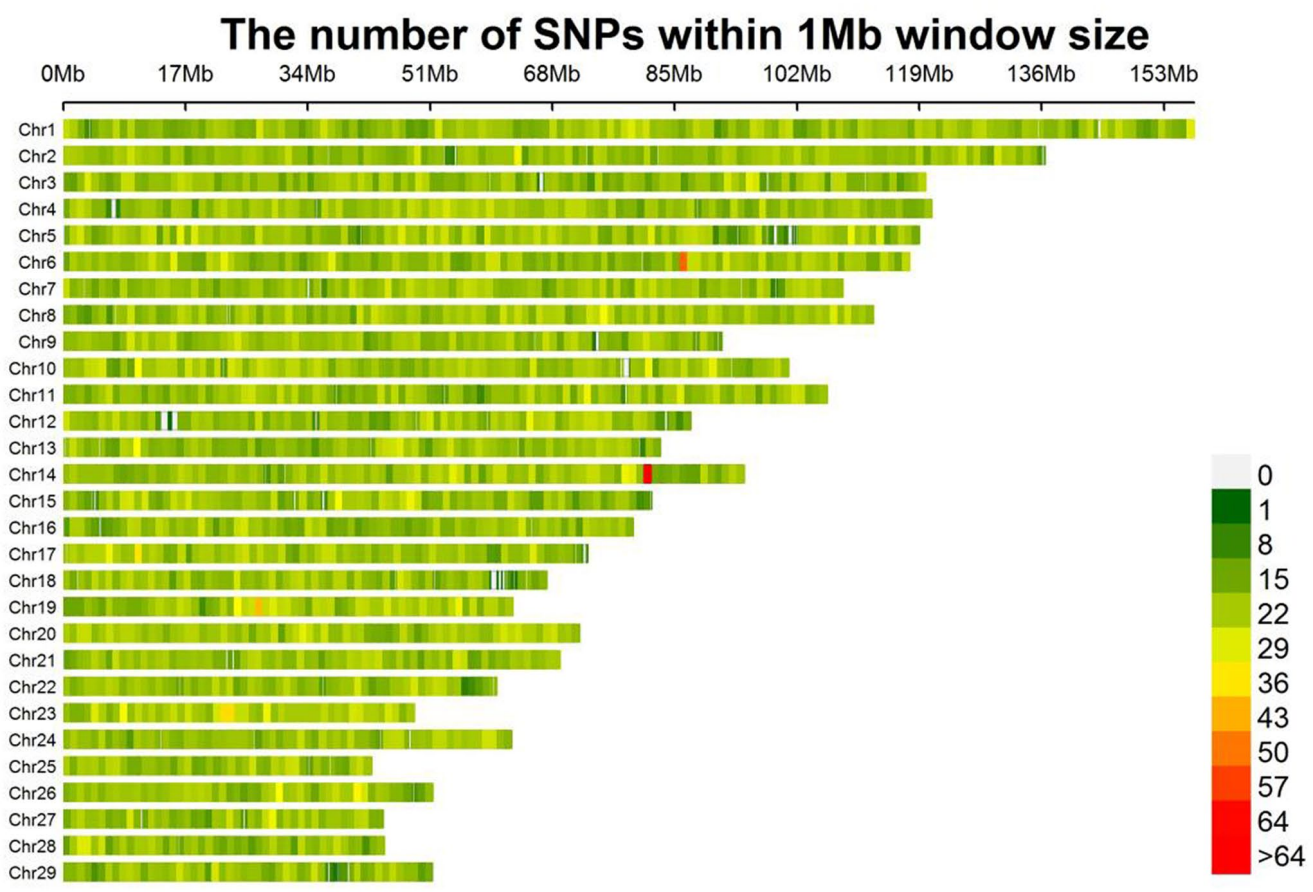
Distribution of SNP density on each chromosome. The figure shows the number of SNPs within 1 Mb window size. As the color changes from green to red, the number of SNPs increases.

The SNPs after quality control are evenly distributed on 29 autosomes in goats (Figure 1). A total of 50,794 SNPs were kept to be used in the next analysis. In this study, a total of four fleece traits were collected, and the descriptive statistics of phenotype data in each fleece trait were presented in Table 2, including the abbreviation of each trait, the number of records (N), the maximum (Max), minimums (Min), mean, standard deviation (SD), and coefficient of variation (CV) values. The average values of four fleece traits in male individuals, including fiber length, cashmere diameter, cashmere length, and cashmere production, are 20.67 cm, 14.91 μm, 6.68 cm, and 1022.26 g, and the corresponding coefficient of variations were 20.46%, 6.44%, 17.66%, and 37.27%, respectively. The average values of four fleece traits in female animals, including fiber length, cashmere diameter, cashmere length, and cashmere production, are 19.27 cm, 15.20 μm, 6.43 cm, and 762.84 g, and the corresponding coefficient variations were 24.08%, 4.87%, 16.49%, and 23.58%, respectively.

**Table 2:** Descriptive statistics of phenotypic values of fleece traits in IMCGs.

Trait	Sex	Number of records	Max	Min	Mean	SD	CV (%)
FL (cm)	Male	372	33	9	20.67	4.23	20.46
	Female	1883	35	6	19.27	4.64	24.08
CD (μm)	Male	369	18.33	12.82	14.91	0.96	6.44
	Female	1880	17.89	12.24	15.20	0.74	4.87
CL (cm)	Male	372	11.0	3.5	6.68	1.18	17.66
	Female	1883	11.0	2.0	6.43	1.06	16.49
CP (g)	Male	370	2,400	118	1022.26	381.00	37.27
	Female	1879	1856	100	762.84	179.91	23.58

Fiber length (FL); Cashmere diameter (CD); Cashmere length (CL); Cashmere production (CP).

### 3.2 Effect of GBLUP and Bayesian methods on the accuracy of GEBV

**Figure 2:**
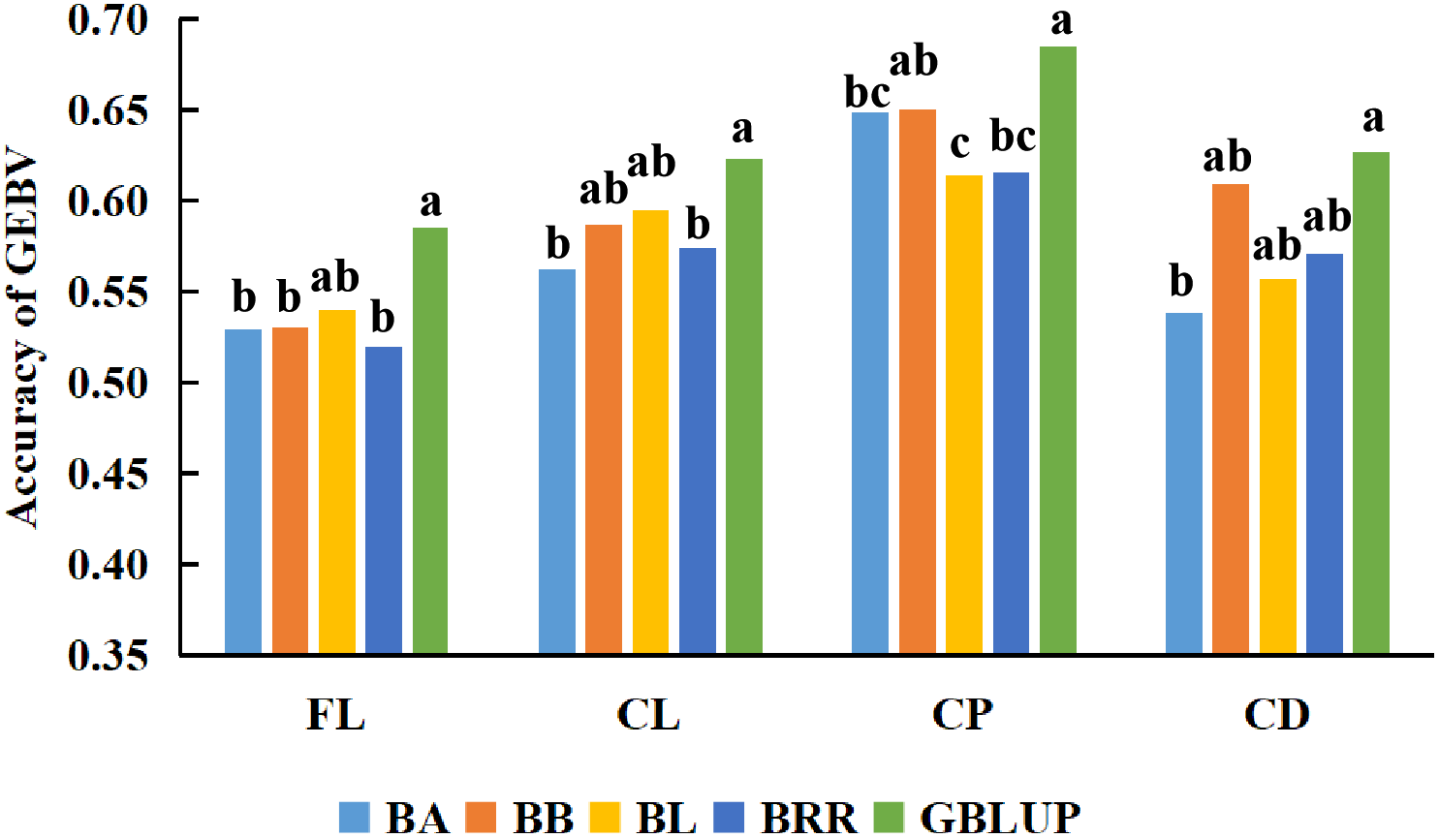
Comparison of the accuracy of GEBV for fleece traits with different methods. The x-axis in the figure represents the different methods used in this study to estimate the genomic breeding values of Inner Mongolia Cashmere Goats fleece traits. The *y*-axis represents the accuracy of estimating the genomic breeding values of fleece traits in Inner Mongolia Cashmere Goats using different methods. The different letters on the graph represent significant differences, while the same letters have no difference.

First, BayesA, BayesB, Bayesian LASSO, BayesRR, and GBLUP methods were used to estimate the genomic breeding value of fleece traits in Inner Mongolia Cashmere Goats. Then, we used the analysis of variance and multiple comparisons to determine the best method for genomic selection in fleece traits of IMCGs. The results of the variance analysis are presented in Table 3. It was shown that methods had a significant effect on the accuracy of genome prediction for cashmere length and cashmere production but had no significant effect on the accuracy of genome prediction for fiber length or cashmere diameter. The multiple comparison results of the accuracy of genome prediction of fleece traits in Inner Mongolia cashmere goats under five methods are shown in Table 4 and Figure 2. The range of genomic predictability of the fleece traits by using the GBLUP, BayesA, BayesB, Bayesian LASSO, and BayesRR methods is 58.52%~68.49%, 52.97%~64.89%, 53.00%~65.04%, 54.01%~61.43%, and 51.95%~61.56%, respectively. It was found that the genomic prediction accuracy with the GBLUP method is better than that with the BayesA, BayesB, Bayesian LASSO, and BayesRR methods. There was no significant difference in prediction accuracy among the Bayes series methods for the fleece traits in Inner Mongolia Cashmere Goats.

**Table 3:** Variance analysis of the impact of methods on the accuracy of GEBV for fleece traits in Inner Mongolia Cashmere Goats.

Trait	Source	DF	SS	MS	*F*	*p*-value
FL	Methods	4	0.0400	0.0100	2.17	>0.05
	Error	70	0.3222	0.0046		
	Corrected total	74	0.3623			
CL	Methods	4	0.0363	0.0091	2.89	<0.05
	Error	70	0.2192	0.0031		
	Corrected total	74	0.2555			
CP	Methods	4	0.0513	0.0128	5.24	<0.01
	Error	70	0.1713	0.0024		
	Corrected total	74	0.2225			
CD	Methods	4	0.0817	0.0204	2.08	>0.05
	Error	70	0.6866	0.0098		
	Corrected total	74	0.7683			

*p* < 0.01: the difference is extremely significant; *p* < 0.05: the difference is significant; *p* > 0.05: the difference is not significant; DF, degree of freedom; SS, sum of square; MS, mean square.

**Table 4:** Accuracy of GEBV in each fleece trait under different methods.

Trait	Bayes A	Bayes B	Bayesian LASSO	BayesianRR	GBLUP
FL	0.5297 ± 0.02^b^	0.5300 ± 0.01^b^	0.5401 ± 0.02^ab^	0.5195 ± 0.02^b^	0.5852 ± 0.02^a^
CL	0.5624 ± 0.01^b^	0.5869 ± 0.01^ab^	0.5950 ± 0.01^ab^	0.5740 ± 0.01^b^	0.6229 ± 0.02^a^
CP	0.6489 ± 0.01^bc^	0.6504 ± 0.01^ab^	0.6143 ± 0.02^c^	0.6156 ± 0.01^bc^	0.6849 ± 0.01^a^
CD	0.5384 ± 0.04^b^	0.6096 ± 0.01^ab^	0.5569 ± 0.03^ab^	0.5703 ± 0.02^ab^	0.6270 ± 0.02^a^

^a,b^Represent significant differences. The difference is significant with different letters.

### 3.3 Effect of reference population size on the accuracy of GEBV

**Figure 3:**
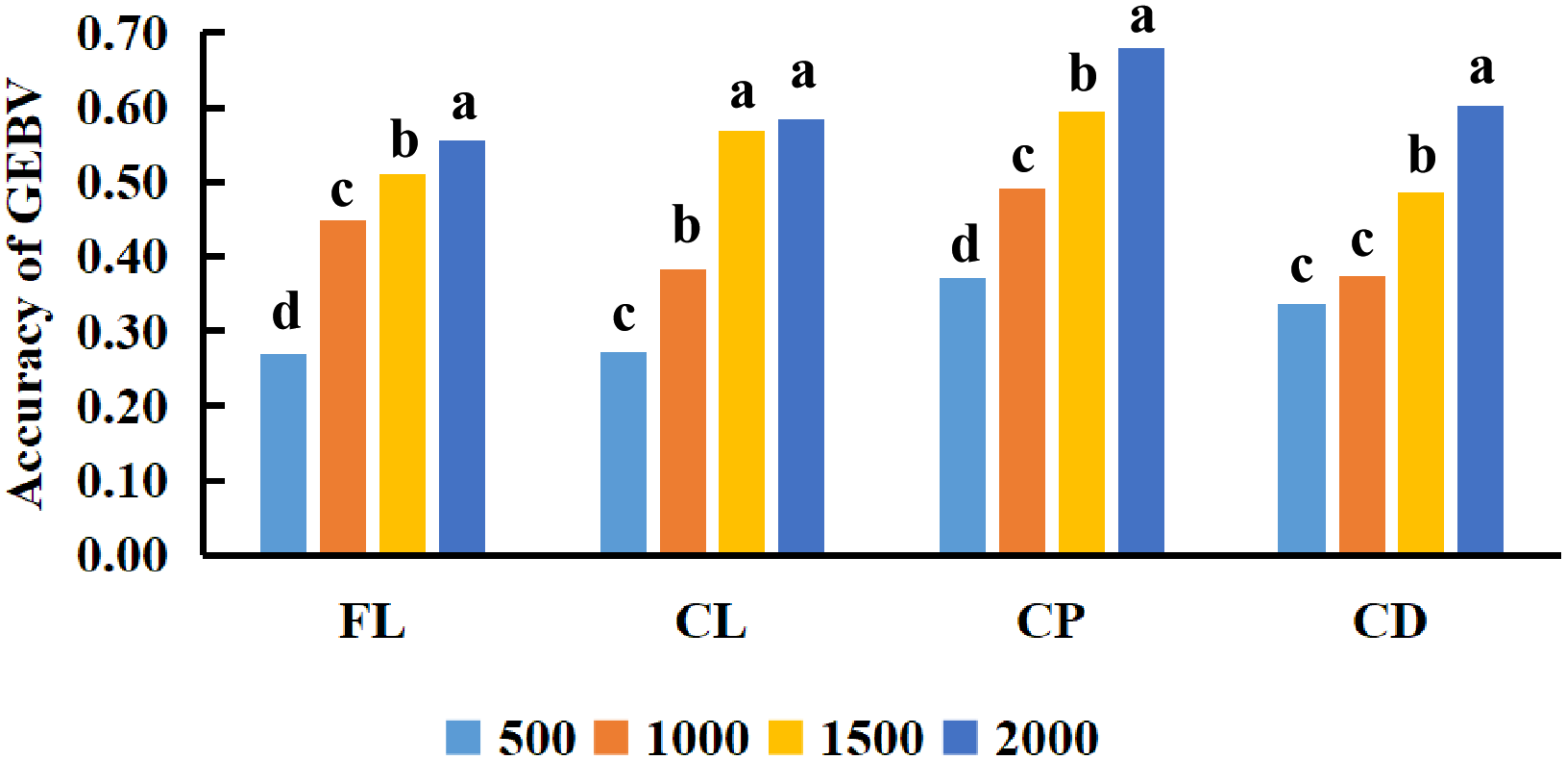
Comparison of the accuracy of GEBV for fleece traits with different reference population sizes. The *x*-axis in the figure represents the different reference population sizes used in this study to estimate the genomic breeding values of Inner Mongolia Cashmere Goats fleece traits. The *y*-axis represents the accuracy of estimating the genomic breeding values of fleece traits in Inner Mongolia Cashmere Goats using different reference population sizes. The different letters on the graph represent significant differences, while the same letters have no difference.

This study also compared the impact of different reference population sizes on the accuracy of estimated genomic breeding values for fleece traits in Inner Mongolia Cashmere Goats. Based on the above results, the GBLUP method is the best method for evaluating the accuracy of genomic selection of fleece traits in Inner Mongolia Cashmere Goats. The reference populations with sizes of 500, 1,000, 1,500, and 2,000 were set to perform genomic selection of fleece traits in IMCGs. The results of the variance analysis of reference population sizes are presented in Table 5. It was shown that reference population size had a significant effect on the accuracy of genomic prediction for fleece traits in IMCGs. The multiple comparison results of the accuracy of genomic prediction of fleece traits under different reference population sizes are shown in Table 6 and Figure 3. For CL traits, when the reference population size is between 1,500 and 2,000, there is no significant difference in the accuracy of the genomic breeding value. However, the accuracy of GEBV with reference population sizes of 1,500 and 2000 is significantly higher than that with 500 and 1,000 reference population sizes. The accuracy of GEBV for CL is 56.91–58.39%. For FL, CP, and CD traits, there was a significant difference between 2,000 and the other three levels (500, 1,000, and 1,500) in the reference population. The accuracy of genomic breeding values of 55.47%, 67.87%, and 60.11% in the reference population was 2,000 for FL, CP, and CD traits, respectively. Therefore, it is necessary that the reference population size be expanded to perform genome selection in IMCGs.

**Table 5:** Variance analysis of the impact of reference population size on the accuracy of GEBV for fleece traits in Inner Mongolia Cashmere Goats.

Trait	Source	DF	SS	MS	F	*p*-value
FL	Methods	3	0.6711	0.2237	97.11	<0.01
	Error	54	0.1244	0.0023		
	Corrected total	57				
CL	Methods	3	0.9491	0.3164	64.54	<0.01
	Error	53	0.2598	0.0049		
	Corrected total	56	1.2089			
CP	Methods	3	0.7621	0.2540	41.03	<0.01
	Error	55	0.3406	0.0062		
	Corrected total	58	1.1027			
CD	Methods	3	0.6297	0.2099	20.42	<0.01
	Error	54	0.5551	0.0103		
	Corrected total	57	1.1849			

*P* < 0.01: the difference is extremely significant; *P* < 0.05: the difference is significant; *P* > 0.05: the difference is not significant; DF, degree of freedom; SS, sum of square; MS, mean square.

**Table 6:** Accuracy of GEBV in each fleece trait under different reference population size levels.

Trait	500	1,000	1,500	2000
FL	0.2702 ± 0.02^**d**^	0.4478 ± 0.02^**c**^	0.5100 ± 0.01^**b**^	0.5547 ± 0.01^**a**^
CL	0.2711 ± 0.01^**c**^	0.3829 ± 0.03^**b**^	0.5691 ± 0.01^**a**^	0.5839 ± 0.01^**a**^
CP	0.3723 ± 0.02^**d**^	0.4906 ± 0.03^**c**^	0.5942 ± 0.02^**b**^	0.6787 ± 0.01^**a**^
CD	0.3360 ± 0.02^**c**^	0.3738 ± 0.03^**c**^	0.4863 ± 0.04^**b**^	0.6011 ± 0.02^**a**^

^a,b^Represent significant differences. The difference is significant with different letters.

## 4 Discussion

In order to effectively apply genomic selection to design the breeding plan for Inner Mongolia Cashmere Goats, it is necessary to determine the factors affecting prediction accuracy. Therefore, we collected the cashmere performance records of 2,255 individuals to investigate the influence of methods and reference population size on the accuracy of genomic prediction.

This study was conducted to compare the genomic prediction ability of fleece traits in IMCGs using the GBLUP and Bayes series methods (BayesA, BayesB, Bayesian LASSO, and Bayesian Ridge Region). It was observed that the methods had a significant effect on the accuracy of genomic prediction for cashmere length and cashmere production. The genomic prediction accuracy with the GBLUP method is better than that with Bayesian methods. This result is also consistent with that of many previous studies. Baby et al. used the GBLUP and BayesB methods to evaluate the genomic estimated breeding values for 16 meat quality traits in the Berkshire population (*n* = 1,191) (26). The results showed that the GEBV accuracy ranged from 0.42 for collagen to 0.75 for water-holding capacity with the GBLUP method. Under the Bayes B model, the GEBV accuracy ranged from 0.10 for the National Pork Producers Council marbling score to 0.76 for drip loss. Zhu et al. (27) used the GBLUP and Bayesian Alphabet models to estimate the genomic breeding values of six wool traits in Alpine Merino sheep. The accuracy of the GBLUP method was slightly higher than that of the Bayesian methods. For the datasets of low-density SNP genotypes, the genomic prediction accuracy of wool traits was 0.34–0.0.60 for GBLUP. For the datasets of high-density SNP genotypes, the genomic prediction accuracy of wool traits was 0.35–0.57 for the GBLUP method. Silva et al. reported the genomic prediction ability for carcass composition indicator traits in Nellore cattle using the BLUP, GBLUP, ssGBLUP, and Bayesian methods (BayesA, BayesB, BayesC, and Bayes LASSO) (28). In terms of predictive ability and bias, it is identical in terms of the visual score trait between the Bayesian and GBLUP methods. However, the accuracy of GEBV with the GBLUP method is higher than that with the BayesB method for carcass traits. Vu et al. evaluated the impact of different prediction methods (BayesA, BayesCπ, and GBLUP) on the accuracy of GEBV in the Portuguese oyster (*Crassostrea angulata*) (29). It was indicated that the accuracy with GBLUP is slightly higher than that with Bayes methods, but there was no significant difference among the methods. The accuracy of genomic predictivity for the traits is 0.240–0.794. With the continuous progress of breeding work, more efficient and simple models will be optimized and developed. Applying these methods to the genomic selection of important traits in livestock and poultry will inevitably accelerate the breeding process of the population.

The size of the reference population is an important factor affecting the accuracy of genomic selection. How to reasonably construct a reference population for genomic selection in IMCGs is important. In this study, different reference population sizes (500, 1,000, 1,500, and 2000) were set to evaluate the accuracy of genomic selection for fleece traits in IMCGs. It was found that the size of the reference population has a significant impact on the accuracy of genomic prediction for fleece traits. Baby et al. reported that the GEBV accuracy increased with the size of the training data. In general, the GEBV accuracy with the Bayes B model was lower than that with the GBLUP model, especially for the small training sample size (26). Uemoto et al. (30) used simulated phenotype data under different scenarios to assess the prediction accuracy of GEBV under population size using a reference-test validation design. It was found that a large population size is needed to increase the accuracy of GEBV. Nwogwugwu et al. assessed genomic prediction ability by using the reference population of 1,000, 2000, 3,000, and 5,000 randomly selected from generations 7, 8, and 9 in a simulated Korean beef cattle population (31). According to the simulation results, the accuracy of genomic selection gradually increases as the number of reference populations increases. Kabanov et al. used three methods to assess breeding value and predictability for five main traits of Large White pigs (32). The research results showed that the accuracy of genomic selection also gradually increases with the size of the reference population. This also indicated that the size of the reference population has a certain impact on the accuracy of genomic selection. When the reference population size reaches a certain level, the accuracy of genomic selection cannot be significantly improved. This is similar to the cashmere length trait. The accuracy of genomic selection in IMCGs between the reference population size of 1,500 and 2000 had no significant difference. Therefore, it is important to choose a reasonable reference population size to perform genomic selection, which can ensure the accuracy of genomic selection while saving costs.

## 5 Conclusion

To summarize, this study used GBLUP and Bayesian methods (BayesA, BayesB, Bayesian LASSO, and Bayesian Ridge Region) to perform the genomic prediction. The 5-fold cross-validation was utilized to evaluate the accuracy of GEBV. It was found that the prediction accuracy for fleece traits in IMCGs with the GBLUP method is the highest. It indicates that the GBLUP method should be used for the genomic selection of Inner Mongolia Cashmere Goats. At the same time, it was demonstrated that the accuracy of genomic prediction for fleece traits with a reference population of 2000 is significantly higher than other scale reference populations. Therefore, it is necessary to further expand the size of the reference population to increase the accuracy of GEBV for fleece traits in Inner Mongolia Cashmere Goats.

## Data availability statement

The original contributions presented in the study are publicly available. This data can be found here: https://db.cngb.org/; CNP0005155.

## Ethics statement

The animal studies were approved by the studies involving animals were reviewed and approved by the Laboratory Animal Welfare and Animal Experiment Ethics Inspection Committee of Inner Mongolia Agricultural University. The studies were conducted in accordance with the local legislation and institutional requirements. Written informed consent was obtained from the owners for the participation of their animals in this study.

## Author contributions

XY: Data curation, Formal analysis, Software, Writing – original draft, Writing – review & editing. JZ: Software, Writing – original draft. JL: Writing – review & editing. NW: Data curation, Writing – original draft. RS: Writing – original draft, Writing – review & editing. ZW: Writing – original draft, Writing – review & editing.
